# Phenol levels during intralesional curettage and local adjuvant treatment of benign and low-grade malignant bone tumours

**DOI:** 10.1186/2045-3329-2-10

**Published:** 2012-03-26

**Authors:** Suzan HM Verdegaal, Jan den Hartigh, Pancras CW Hogendoorn, Hugo FG Brouwers, Antonie HM Taminiau

**Affiliations:** 1 Department of Orthopaedic Surgery, Leiden University Medical Center, Leiden, The Netherlands; 2 Department of Clinical Pharmacy and Toxicology, Leiden University Medical Center, Leiden, The Netherlands; 3 Department of Pathology, Leiden University Medical Center, Leiden, The Netherlands

## Abstract

**Background:**

Phenol is widely used for years as local adjuvant treatment for bone tumours. Despite its use for a long time, no information is available about the local concentration of phenol that is achieved in an individual patient, and the most sufficient and safe procedure to wash out the phenol after using it as local adjuvant.

**Questions/purposes:**

1. What is the initial local concentration of phenol in the tissue of the cavity wall after the application of phenol? 2. How quickly is phenol 85% diluted by washing the bone cavity with ethanol 96% solution? 3. Is the degree and speed of dilution influenced by the size of the cavity? 4. How many times should the cavity be rinsed to obtain sufficient elimination of phenol?

**Methods:**

A basic science study was performed at respectively 16 and 10 patients, treated by intralesional curettage and adjuvant therapy for low-grade central chondrosarcoma of bone. Test 1:in 16 patients ten samples were collected of the mixture of phenol and ethanol from the bone cavity. Test 2:in ten patients, two biopsy samples were taken from the cavity wall in the bone during surgery.

**Results:**

Phenol concentrations had wide variety in different patients, but all decreased by rinsing with ethanol.

**Conclusions:**

Ethanol 96% is effective to wash out local applicated phenol, by rinsing the bone cavity six times. The local concentration of phenol diminishes to an acceptable concentration of 0.2%. This study provides new insights to safely further improve the surgical technique of intralesional treatment of bone tumours.

## Background

In the surgical treatment of benign bone tumours, intralesional curettage has been performed throughout the past century. Over the recent years, **its **use has been extended to low-grade intramedullary malignant tumours in some instances. Unfortunately, this surgical technique has the risk of local recurrence from tumour cells that may be left behind. For this reason curettage was supplemented by the use of a local adjuvant, such as phenol, liquid nitrogen or bone cement (poly methyl methacrylate, PMMA). Various studies have demonstrated that by using adjuvant, the results of local therapy have been greatly improved [1-4].

With regard to liquefied phenol, only a few documented studies have been published, despite of **its **routine use over a long period in orthopaedic practice. Previously, we were able to show that in case of low-grade chondrosarcoma, phenol is able to kill tumour cells *in-vitro *already at a concentration of 3% [5]. In clinical settings, liquefied phenol is known to reduce local recurrence rates in different benign bone tumours such as aneurysmal bone cysts, chondroblastoma and giant cell tumours from **41% to 7-9% **[1,2]. Liquefied phenol, containing 82.0 to 86.5% w/w phenol in water, is a colourless or faintly coloured liquid. It may be used as a preservative in pharmaceuticals and chemicals, but is also widely used in household products. Liquefied phenol is readily absorbed via inhalation, ingestion and dermal contact, causing both local and systemic toxicity. The elimination half-life ranges from 1-14 hours [6]. It is eliminated in the urine, mainly as sulfate and glucuronide conjugates. Liquefied phenol causes cell-wall disruption, precipitation, denaturation of proteins and coagulation necrosis. Aqueous solutions as dilute as 10% may be corrosive.

Clinical symptoms of phenol after ingestion may include local corrosion with pain, nausea, vomiting and diarrhea [6]. Systemic toxicity may consist of CNS depression, circulatory and respiratory failure, pulmonary edema and hepatic and renal injury. Poisoning may occur from skin contact, especially from wounds. Applied to the skin, phenol causes blanching and corrosion in a concentration of 1-2%, depending on the exposure time.

In case of surgery, a bone-window is created in order to perform a subsequent curettage of the tumour. To ensure that the cavity that has been formed is microscopically tumour-free, the walls are cauterised with an 85% solution of liquefied phenol. Given that liquefied phenol has toxic and potentially carcinogenic properties when used systemically over a longer period, the cavity is then rinsed a number of times with a 96% solution of ethanol. The cavity is filled with allograft bone-chips and the bone-window, which has also been rinsed with liquefied phenol and ethanol, is placed back in position. The above mentioned surgical technique has our preference given its advantage to induce minimal local damage and a biological reconstruction. Alternative products to fill the resultant defect after curettage are PMMA, auto graft bone chips or synthetic bone materials.

Regarding the surgical technique, some questions remain:

1. What is the initial local concentration of phenol in the tissue of the cavity wall after the application of liquefied phenol 85%?

2. How quickly is the liquefied phenol 85% diluted by washing the bone cavity with ethanol 96% solution?

3. Is the degree and speed of dilution influenced by the size of the cavity?

4. How many times should the cavity be rinsed to obtain sufficient elimination of phenol?

Answering these questions will further elucidate the safe practice of phenol-assisted, intralesional curettage of low grade malignant intramedullary bone tumours.

## Patients and methods

### Patients

16 patients (5 male, 11 female) with a median age of 48 (range 26-70) at time of surgery, were treated for grade I chondrosarcoma. The tumours are predominantly located in the proximal humerus (7/16, 44%) and the distal femur (5/16, 31%) (Table 1). The lesions were histologically classified by an experienced pathologist according to the recently published consensus criteria [7] and graded according to the Evans' grading [8].

**Table 1:** Patient information, tumour volume and cavity surface

Patient	Age/Gender	PA	Location	Tumour volume (cm3)	Cavity surface (cm2)
1	41/M	CHS I	Proximal humerus	27	73,4
2	39/M	CHS I	Distal femur	48,1	74,2
3	40/F	CHS I	Distal tibia	9,2	24,6
4	411 M	CHS I	Proximal humerus	26,3	62,3
*5*	*45/F*	CHS I	Proximal humerus	40,8	72,6
6	70ff	CHS I	Distal femur	10,9	62,3
7	*55/M*	CHS I	Distal femur	39,4	74,6
8	59/F	CHS I	Proximalhumerus	14,7	33,4
9	49/F	CHS II	Proximal humerus	9,7	25,8
10	48/F	CHS I	Distal femur	33	58,8
11	67/F	CHS I	Distal tibia	*5,2*	16,8
12	44/F	CHS I	Proximal humerus	25,8	49,4
13	33/NI	CHS I	Metacarpal	V 4,4	15,3
14	50/F	CHS I	Humerus	44	74,5
*15*	*55/F*	CHS I	Proximal humerus	32	*59,3*
16	26/F	CHS I	Distal femur	20	*51,5*

Characteristics of the 16 patients where the tests during surgery were performed. Depending on the location of the chondrosarcoma, the cavity surface varies.

### Clinical setting

Following a trial on three patients to test the feasibility of the study, 16 patients, suspected with grade I chondrosarcoma following X-ray and dynamic Gd-MRI [9], were treated according to the surgical technique described above. Two tests were performed. Test 1 was done on all 16 patients, test 2 on 10 patients. Before performing surgery including these tests, all patients were informed and gave their approval. Patient material was used in a coded fashion according to the local ethical regulation and in accordance to the national ethical guidelines (Dutch organisation of scientific societies FEDERA; "Code for proper secondary use of human tissue" in the Netherlands).

### Treatment protocol

#### Test 1

Depending on the size of the cavity, 2 to 4 ml of liquefied phenol 85% was applied with one or more small gauzes at the inner surface of the wall for 3 minutes after curettage of the bone tumour. Then the cavity was filled with an ethanol 96% solution. The volume of ethanol differed depending on the size of the cavity. Subsequently, this mixture of phenol and ethanol was extracted by syringe and sealed immediately in a polypropylene container. This procedure was repeated ten times and labelled accordingly (sample numbers I to X).

#### Test 2

During surgery two biopsies were taken. The first biopsy (A)was taken after the cavity had been swabbed with the 85% phenol solution. A second biopsy (B) was taken after the cavity had been rinsed thoroughly ten times with ethanol, as described above (Table 2).

**Table 2:** Concentrations of phenol in sixteen patients during washing out phenol with ethanol

	1	2	3	4	5	6	7	8	9	10	11	12	13	14	15	16
I	929	2435	8032	3264	9661	13000	289	1300	1500	1500	1500	2299	20974	1239	373	160
II	449	900	2889	730	1780	563	193	787	734	1500	1478	816	13932	889	247	118
III	177	469	2169	293	1097	201	555	473	329	539	1199	523	4652	644	159	97
IV	92	313	1019	83	944	119	156	418	249	195	495	292	1739	465	82	66
V	84	185	797	106	632	162	167	313	150	160	185	261	1932	407	77	89
VI	69	189	522	73	312	126	146	289	127	87	249	223	1287	307	82	54
VII	65	128	400	49	168	101	98	191	97	126	157	160	805	214	65	64
VIII	65	146	381	72	227	72	114	126	97	116	217	140	900	215	65	39
IX	42	325	294	59	392	138	52	124	110	57	178	111	597	162	87	42
X	105	217	279	47	200	54	64	98	88	60	143	117	417	182	60	29

Results of 16 patients (1-16); after using phenol 85% as adjuvant therapy, ethanol 96% was used to wash out the phenol; sample I to X show ppm of phenol in ethanol; Note that the bold results are not reliable or higher measured level than in reality.

These biopsies (A and B), plus the ten flush solutions (I to X) were then further investigated to determine the concentration of phenol.

### The surface area of the cavity

It is necessary to approximate the shape of the cavity, because the actual surface area does not fit in any standard mathematical model and therefore cannot be measured with absolute precision. Therefore we projected a cylinder over the lesion using the largest diameter of the cartilaginous tumour of the long bones, measured in three dimensions on the pre-operative MRI. A standard formula was used to calculate the inner surface of this cylindrical curetted bone cavity: *2 πr (r + h). R *represents the average of half the width (mediolateral) and half the depth (anteroposterior), which often corresponds with the inside diameter of the bone. *H *is the length of the tumour taken in a craniocaudal direction.

### Sampling

The flush solutions, from test 1, and the bone biopsies, from test 2, were stored in a refrigerator until analysis. Flush solutions were analyzed as such, or after dilution (see phenol analysis). Bone biopsies were dispersed in 5.0 ml ethanol 96% v/v; an aliquot of this solution was analyzed.

### Phenol analysis

The phenol concentration of the ethanolic flush solutions was determined by High Performance Liquid Chromatography (HPLC) with spectrophotometric detection. Briefly, chromatographic analysis of the samples (diluted with ethanol to fit within the concentration range of the calibration curve, if necessary) was performed on a silica reversed phase column (Nucleosil C18, 100 × 3 mm, 5 μm particle size) with a mobile phase consisting of 25 mM phosphate buffer containing 0,5% triethylamine pH 3.0 and acetonitrile (83 + 17, v/v). The column flow rate was 0.4 ml/min and detection was performed at 212 nm. The injection volume was 10 μl. The phenol concentrations in the flush solutions were calculated from calibration graphs obtained by simultaneous analysis of phenol standard solutions in ethanol 96% v/v in the concentration range 30 - 1500 ppm phenol. Up to 500 ppm there was a linear relation between response and concentration; above 500 ppm a quadratic function had to be used. Inter-day reproducibility showed a coefficient of variation of 5.1% at a concentration level of 50 ppm and 10.6% at 5 ppm (n = 6). The lower limit of quantitation (LLOQ) was 5 ppm. Basic descriptive statistics were employed.

## Results

### Test 1

**Figure 1:**
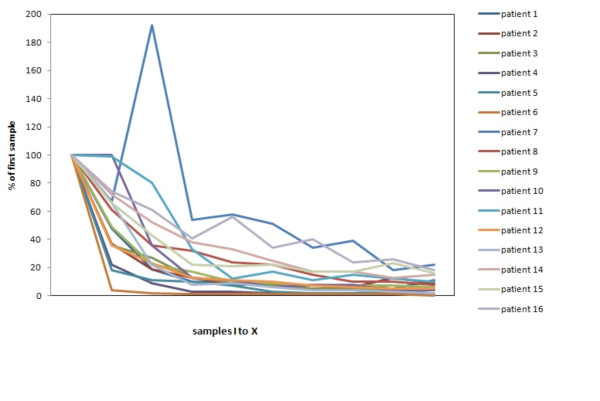
**Concentrations of phenol in ethanol**. Results of ten samples of ethanol with phenol in 16 patients; samples II to X are reproduced as the fraction of the phenol concentration, measured in the first sample.

The phenol concentrations measured in the flush solutions ranged from 29 to 20974 ppm. Large inter-patient differences were observed. Figure 1 shows a graphical presentation of the decay in phenol concentration over time observed for each patient, expressed as a percentage of the concentration measured in the first flush sample.

After washing the bone cavity five times with ethanol 96%, in most of the patients the phenol levels measured are < 280 ppm.

Patient number 7 has one very high concentration of phenol in sample III (Table 2). We can only explain this as a sample error, because from sample IV to X the concentration decreases.

### Test 2

In Table 3 the phenol concentrations found in the biopsies, dispersed in 5 ml. ethanol, are presented. For all patients the phenol concentration before (A) and after flushing the cavity with ethanol 96% (B) is shown. Remarkable differences were measured, quantative as well as in reducing the concentration of phenol washed out by ethanol.

**Table 3:** Concentrations of phenol solved in 5 ml. of ethanol during surgery

	8	9	10	11	12	13	14	15	16
biopsy A (ppm)	1598	888	168	479	1043	25	572	94	144
biopsy B (ppm)	408	418	39	106	636	17	587	11	18

Results of phenol samples 9 patients (8-16); first bone biopsy (A) was taken after application phenol 85%. After washing ten times with ethanol 85% the second biopsy (B) was performed. **The concentrations of phenol (ppm) in this table are solved in 5 ml. of ethanol**.

### Tumour volume

Median volume of the cavity is 24.4 cm3 (range 4-48), and depends on the location of the tumour. The smallest volumes are measured in the distal tibia and fifth metacarpal bone.

**Figure 2:**
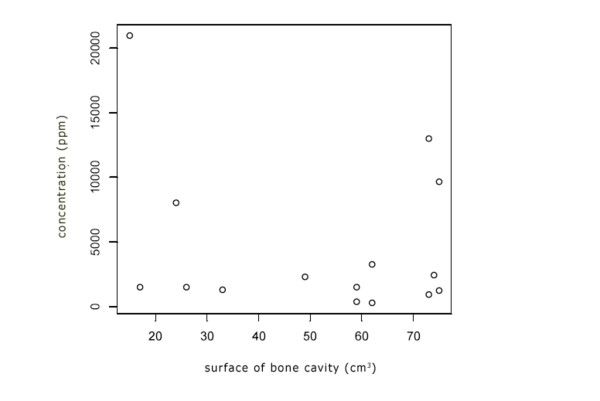
**Box Plot of concentration phenol and surface of bone cavity**. Relation between concentrations of phenol measured in ethanol (ppm) and surface of the bone cavity. No correlation is seen between the concentrations of phenol in ethanol in large or small cavities.

No correlation is seen between the concentrations of phenol in ethanol in large or small cavities (Figure 2).

## Discussion

The use of chemical cauterization for benign and low-grade malign bone tumours was first proposed by Bloodgood [10]. Although phenol is used for many years as adjuvant therapy in the intralesional treatment of benign and low-grade malignant bone tumours, no information is available about the local concentration of phenol in individuals, the most sufficient and safest procedure to wash out the phenol after the local use.

Some surgeons have a dislike to the use of phenol as adjuvant, due to the possible predisposition to infection, the inhibition of the incorporation of grafts, systemic toxic effects on patients, and possible adverse effects on personnel in the operating theatre.

In our opinion, the highest estimated risk for using phenol is the people who work with it in the operating room. To prevent skin contact, using the routine measures such as by wearing proper protection to hands and face is sufficient. In case of skin contact, the skin should be irrigated with polyethylene glycol (PEG) 400 solution, isopropanol 70% or, if not available, with water [10-14]. During surgery, there is a risk of fire, with the evaporation of ethanol and simultaneous use of electric cauterization devices, as this can cause a spark. Therefore, we strongly recommend disconnecting the electric devices from the moment phenol and ethanol bottles are to be opened.

The two biopsies A and B were taken from the cavity wall before starting washing out and after rinsing 10 times with ethanol 96%. In biopsy B, the quantity of phenol is diminished, as expected, but still there is quite a concentration left. This is due to the fact, that phenol is very lipophylic and therefore not easy to remove with ethanol 96%. The results also show remarkable differences in a quantative way. In these patients, tumour volume, differences in dilution by wound fluid and blood in the cavity, and differences in the ml of phenol applicated explain these results. **In patient 14, the concentration even increases a little bit after washing out with ethanol. In this patient, it seems that it is hard to wash out the local phenol from the cavity wall**.

Concerning the initial local concentration of phenol in the cavity wall after application of phenol, two biopsies were curetted. Test 2 shows the concentrations in biopsy A and B. Starting with 85% of liquefied phenol, the mean initial local concentration of phenol measured was 557 ppm (range 25-1598, biopsy A, Table 3). The measurements have a large variation.

Patient 13 suffered from a small chondrosarcoma of the metacarpal bone. The volume of the tumour was 4,4 cm^3^. Biopsy A and B show 25 and 17 ppm phenol respectively. Nevertheless, the first sample in test 1 has a very high concentration of phenol in ethanol, 20974 ppm. The possible explanation of these controversial measurements is, that the location where the biopsy A and B were taken from the metacarpal bone can almost only be cortical border of the tumour. As cortical bone won't absorb phenol very well, the concentrations in biopsy A and B are low.

To investigate the effect of ethanol to dilute the applicated phenol, 10 samples in 16 patients were collected. In test 1, in all 16 cases the highest concentration of phenol was measured in the first sample. However, the absolute values differ. Despite the fact that the same procedure was performed in all cases, no procedure is the same given the different volumes of the tumours varying from 4 to 48 cm^3^. In patient number seven, the III^rd ^sample shows an increase of the concentration phenol. From the IV^th ^sample, the line decrease further on sample II. The III^rd ^sample must be considered to be a sample error. In these tests, the cavity was filled with ethanol ten times, and phenol concentrations were measured. After washing the cavity 6 times, in 10 of 16 patients the fraction of the initial concentration of phenol is < 10%. The quantative concentration of phenol is < 1300 ppm in all cases, which means an estimated concentration of phenol of < 0,2%.

The degree of dilution is not influenced by the size of the cavity after curettage. However, it is remarkable that the highest concentrations in the first samples of test 1 all concerned small tumour volumes. This can be explained by the relatively small volume of ethanol where the phenol is diluted in.

We did not specifically study systemic effects of phenol exposure in our patients; however, on retrospective review, no adverse effects or secondary malignancies were noted.

In the few studies that described measurements of phenol, this was done by taking samples of urine, as liquefied phenol is eliminated by the kidneys. However, it is hard to define a 'thresh hold' for the toxic level.

This study shows that the adverse effects on the whole body due to the use of liquefied phenol as adjuvant in the intralesional curettage of benign and low-grade malignant bone tumours are reduced to safe concentrations by washing phenol out by ethanol. Phenol is a safe adjuvant when used in a proper way, taking the above mentioned statements into account. Washing the cavity six times with ethanol 96% will be sufficient to diminish the local concentration of liquefied phenol to an acceptable concentration of < 0.2%.

## Conflict of interests

The authors declare that they have no competing interests.

## Authors' contributions

SHMV participated in the conception and design of the study, performed samples during surgery, performed analysis and interpretation of the data, and drafted the paper. JdH participated in the conception and design of the study, the technical design of the phenol analysis including performing of the laboratory tests, and interpretation of the data. HFGB contributed in acquisition and interpretation of data, and performing the tables and figures. PCWH participated in the conception and design of the study, the analysis and interpretation of the data. AHMT participated in the conception and design of the study, performed the surgery and took samples during surgery and performed analysis and interpretation of the data. All authors read and approved the final manuscript.
